# Linoleic Acid Stimulates [Ca^2+^]i Increase in Rat Pancreatic Beta-Cells through Both Membrane Receptor- and Intracellular Metabolite-Mediated Pathways

**DOI:** 10.1371/journal.pone.0060255

**Published:** 2013-04-02

**Authors:** Yufeng Zhao, Li Wang, Jianhua Qiu, Dingjun Zha, Qiang Sun, Chen Chen

**Affiliations:** 1 School of Biomedical Sciences, The University of Queensland, Brisbane, Queensland, Australia; 2 Department of Otolaryngology, Xijing Hospital, Fourth Military Medical University, Xi’an, China; 3 The Second Affiliated Hospital of Medical School, Xi’an Jiao Tong University, Xi’an, China; Indiana University School of Medicine, United States of America

## Abstract

The role of the free fatty acid (FFA) receptor and the intracellular metabolites of linoleic acid (LA) in LA-stimulated increase in cytosolic free calcium concentration ([Ca^2+^]i) was investigated. [Ca^2+^]i was measured using Fura-2 as indicator in rat pancreatic β-cells in primary culture. LA (20 µM for 2 min) stimulated a transient peak increase followed by a minor plateau increase in [Ca^2+^]i. Elongation of LA stimulation up to 10 min induced a strong and long-lasting elevation in [Ca^2+^]i. Activation of FFA receptors by the non-metabolic agonist GW9508 (40 µM for 10 min) resulted in an increase in [Ca^2+^]i similar to that of 2-min LA treatment. Inhibition of acyl-CoA synthetases by Triacsin C suppressed the strong and long-lasting increase in [Ca^2+^]i. The increase in [Ca^2+^]i induced by 2 min LA or GW9508 were fully eliminated by exhaustion of endoplasmic reticulum (ER) Ca^2+^ stores or by inhibition of phospholipase C (PLC). Removal of extracellular Ca^2+^ did not influence the transient peak increase in [Ca^2+^]i stimulated by 2 min LA or GW9508. The strong and long-lasting increase in [Ca^2+^]i induced by 10 min LA was only partially suppressed by extracellular Ca^2+^ removal or thapsigargin pretreatment, whereas remaining elevation in [Ca^2+^]i was eliminated after exhaustion of mitochondrial Ca^2+^ using triphenyltin. In conclusion, LA stimulates Ca^2+^ release from ER through activation of the FFA receptor coupled to PLC and mobilizes mitochondrial Ca^2+^ by intracellular metabolites in β-cells.

## Introduction

Long-chain free fatty acids (FFAs) diversely regulate pancreatic β-cell function under different conditions. FFAs acutely potentiate glucose-stimulated insulin secretion from both β-cell lines and β-cells in primary culture. On the other hand, they inhibit glucose-stimulated insulin secretion and induce β-cell apoptosis in a long term action on β-cells [1]–[6]. It is well accepted that the effects of FFAs attribute to their intracellular metabolism to synthesize long chain acyl-CoA esters. Long chain acyl-CoA activates or modulates various processes, such as diacylglycerol generation, triglyceride generation, PKC activation and protein acylation, in β-cells to influence insulin secretion [7]–[9]. Acyl-CoA is also transported into mitochondria for β-oxidation, which links fuel metabolism of β-cells to insulin secretion. The discovery of FFA receptors such as GPR40 shows another signaling pathway of FFAs in regulating β-cell function. GPR40 is one of the G-protein coupled receptors that distributed on the plasma membrane of β-cells, and long-chain FFAs are ligands to activate the receptor [10]. It was reported that FFAs activate GPR40 to stimulate insulin secretion from an insulinoma β-cell line, MIN6 cells, and from rodent pancreatic islets [11]–[13]. Therefore, FFAs regulate β-cell function via both intracellular metabolites- and membrane receptor GPR40-mediated pathways.

Insulin secretion is controlled by the levels of cytosolic Ca^2+^ concentration ([Ca^2+^]i). The elevation of [Ca^2+^]i in β-cells, which may be achieved by either calcium influx through membrane channels or calcium release from intracellular calcium stores, triggers and amplifies the exocytosis of insulin granules. Both intracellular metabolites of FFAs and activation of GPR40 may regulate β-cell function via modulating levels of [Ca^2+^]i in β-cell. FFA metabolites, particularly long-chain acyl-CoA, have stimulatory effects on Ca^2+^ release from endoplasmic/sarcoplasmic reticulum in muscle cells [14]. In addition, long-chain acyl-CoA induces mitochondrial permeability transition pore (PTP) formation leading to cell apoptosis of liver cells [15]. The Ca^2+^-mobilizing effects of long-chain acyl-CoA or other metabolites of FFAs in β-cells need to be clarified. On the other hand, GPR40 activation stimulated by FFAs leads to an acute increase in [Ca^2+^]i in β-cells. GPR40 activation by FFAs activates phospholipase C (PLC) to produce inositol triphosphate (InsP3) and the increase in [Ca^2+^]i through Ca^2+^ release from IP3-senstive intracellular Ca^2+^ pools [13], [16]. The relative contribution and significance of this two signaling pathways in FFAs-induced increase in [Ca^2+^]i in β-cells is unknown.

In the present study, we used linoleic acid to observe the effects of FFAs on [Ca^2+^]i levels in primary cultured rat β-cells. The respective effects of the FFA receptor signaling pathway and the intracellular FFA metabolite signaling pathway on [Ca^2+^]i were carefully dissected and the multiple pathways for increase in [Ca^2+^]i were demonstrated.

## Materials and Methods

### Ethic Statement

Sprague-Dawley rats (10–12 weeks old) were purchased from the Animal House of the University of Queensland (UQ). The animal experiment was reviewed and approved by the Animal Care and Use Committee of UQ. The experiment was performed in compliance with the Animal Welfare Act and the guide to the care and use of laboratory animals in UQ. Every effort was made to alleviate animal discomfort and CO_2_ inhalation was applied as the appropriate method of sacrifice.

### Chemicals

Linoleic acid was purchased from Sigma (St. Louis, USA). Histopaque-1077, Dispase, Collagenase (type V), Deoxyribonuclease I (DNase I), Bovine Serum Albumin (BSA), RPMI-1640, Triacsin C, etomoxir, triphenyltin, thapsigargin and all reagents for bath solution were also purchased from Sigma. GW9508 was a gift from GlaxoSmithKline (Hertfordshire, United Kingdom). U73122 was obtained from Calbiochem (San Diego, USA). Fura-2/AM was purchased from Invitrogen (Carlsbad, USA). Fetal calf serum, HEPES and penicillin/streptomycin were from Gibco (Birmingham, USA).

### Preparation and Culture of Rat Pancreatic β-cells

Pancreatic islets were isolated from 10–12 week-old male Sprague-Dawley rats as previously described [17]. Briefly, rats were killed by CO2 inhalation and the pancreas of each rat was inflated by injecting 10 ml collagenase solution into it through the bile duct. The collagenase solution was composed of 0.5 mg/ml collagenase, 0.1 mg/ml DNase I and 1 mg/ml BSA in Hank’s Balanced Salt Solution (HBSS). The pancreases were collected and digested at 37°C for 30 minutes and then were dispersed by shaking. The islets were separated by Histopaque-1077 density gradient centrifugation and collected for cell isolation. The islets were dispersed into single cells by digestion with dispase solution. Dispase solution was composed of 1 mg/ml dispase, 0.1 mg/ml DNase I and 1 mg/ml BSA in Ca^2+^-free HBSS. The islet cells were plated onto glass cover slips coated with 0.01% poly-L-lysine and then cultured at 37°C in RPMI-1640 medium supplemented with 10% heat-inactivated fetal calf serum, 100 IU/ml penicillin and 100 μg/ml streptomycin in a humidified atmosphere of 95% air and 5% CO_2_. The culture medium was changed every 2 days. The cells were used for [Ca^2+^]i recording during day 3–6 in culture.

### Measurement of [Ca^2+^]_i_

Islet cells were loaded with 1 μM Fura-2/AM in RPMI-1640 medium for 30 minutes at 37°C. Cells were subsequently rinsed with bath solution and kept for 20 minutes in this solution to allow full deesterification of the dye. Fura-2 was alternately excited by ultraviolet light at 340 nm and 380 nm wavelength respectively (100 ms exposure and 2-seconds cycle) under the control of Dual-Wavelength Photometry Controller (SDR, Australia). The emission at 510 nm was detected by a photomultiplier tube (PMT model 77348, Oriel, USA). The signal was transmitted into DigiData and recorded by Axoscope 8.2 (Axon Instrument, USA). [Ca^2+^]i was calculated according to the formula described by Grynkiewicz et al [18]. [Ca^2+^]i(nM) =  Kd×(Fo/Fs)×(R-Rmin)/(Rmax-R), where Kd = the Fura-2 disassociation constant (225 nM), Fo = the 380 nm fluorescence in the absence of Ca^2+^, Fs = the 380 nm fluorescence with saturating Ca^2+^, R = the 340/380 nm fluorescence ratio, Rmax = the 340/380 nm ratio with saturating Ca^2+^, and Rmin =  the 340/380 nm ratio in the absence of Ca^2+^. Fo/Fs, Rmax and Rmin were determined in the recorded cells. Briefly, the cells were permeabilized by 20µM ionomycin for 10 min to allow sufficient extracellular Ca^2+^ entry, and the resulting 340/380 nm ratio is Rmax. After a steady value of Rmax had been obtained, the Rmin value was determined by chelating Ca^2+^ with 8 mM EGTA. In the experiments, cells were constantly perfused at a rate of 3 ml/min. Reagents were dissolved in the bath solution just before the recordings and delivered through the perfusion. The β-cells were identified by the response to 16 mM glucose stimulation with the increase in [Ca^2+^]_i_. The experiments of [Ca^2+^]_i_ measurements were done after 15 min recovery from 16 mM glucose stimulation. In our preliminary experiment, the effects of LA, oleic acid, linolenic acid, and palmitic acid on increase in [Ca^2+^]i in beta-cells were observed and they showed same effects on increase in [Ca^2+^]i in beta-cells without difference. We choose LA as representative one for mechanism study in this study because LA has been shown clearly and convincingly to activate GPR40 and easy to use in experiment. It has been demonstrated that the EC50 of LA to activate GPR40 is 9.5µM [19]. Most studies observed the effects of GPR40 activation using LA at 10–100µM. We selected to use LA at 20µM in this study, which is within the dose range of most FFA experiments and produced similar results in variable batches of cells in this experiment. The bath solution used for [Ca^2+^]_i_ measurements was composed of (mM): 140 NaCl, 4.7 KCl, 2.6 CaCl_2_, 1.2 MgSO_4_, 1 NaHCO_3_, 1.2 Na_2_HPO_4_, 3 Glucose and 5 HEPES (pH = 7.4 with NaOH).

### Statistical Analysis

The data are represented as Mean ± SEM for each group. One-way ANOVA was used to analyze the statistical significance between different groups in the [Ca^2+^]_i_ levels. P<0.05 was taken as the minimum level of statistical significance.

## Results

### 1. Linoleic Acid (LA) Stimulated Increase in [Ca^2+^]i in Rat β-cells

**Figure 1:**
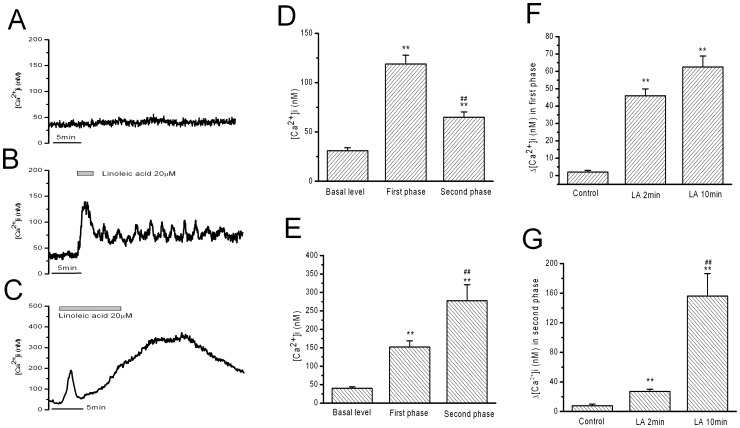
Linoleic acid (LA) stimulated increase in [Ca^2+^]i in rat β-cells. A: blank control. B: Increase in [Ca^2+^]i induced by 2 min LA treatment. C: Increase in [Ca^2+^]i induced by 10 min LA treatment. D and E: the statistical results of the maximal Ca^2+^ levels in the first phase and in the second phase of B and C, respectively. ** means P<0.01 vs basal level, ## means P<0.01 vs the first phase. F: The mean Ca^2+^ change in the first phase. ** means P<0.01 vs blank control. G: The mean Ca^2+^ change in the second phase. ** means P<0.01 vs blank control, ## means P<0.01 vs LA for 2 minutes. (n = 6, 12 and 16 for blank control, 2-min LA and 10-min LA, respectively).

The resting levels of [Ca^2+^]i under 3 mM glucose in rat β-cells remained stable at 46±7 nM during 40 min recording (Fig. 1A, n = 6). A short stimulation by LA (20 µM for 2 min) stimulated a rapid and transient peak increase in [Ca^2+^]i followed by a minor plateau increase in [Ca^2+^]i for more than 10 min (Fig. 1B and D, n = 11). The transient peak increase in [Ca^2+^]i has a maximal amplitude of 119±9 nM and a duration of 3.4±0.23 minutes (Fig. 1F, n = 11). The maximal amplitude of the plateau increase was 65±5 nM for more than 10 min (Fig. 1F, n = 11). When stimulation time was elongated to 10 min for 20 µM LA, rat β-cells exhibited the transient peak increase followed by a strong and long-lasting increase in [Ca^2+^]i. The maximal amplitude of the first phase of peak increase was 152±17 nM for a duration of 3.4±0.18 min, which were not different to those obtained by 2 min LA stimulation. The strong and long-lasting second phase increase in [Ca^2+^]i was however significantly higher than the plateau increase obtained by 2 min LA stimulation, with a maximal amplitude of increase in [Ca^2+^]i as 277±43 nM (Fig. 1C and E, n = 16). The strong and long-lasting increase was recovered slowly for more than 10 min after removal of LA (Fig. 1B).

The mean [Ca^2+^]i change during 3.5 min from start of LA stimulation (first phase increase in [Ca^2+^]i) was not significantly different between 2 min LA stimulation and 10 min LA stimulation (Fig. 1F). The mean Ca^2+^ change in the following 10 min from 3.5 min time point was significantly smaller in 2 min LA stimulation than in 10 min LA stimulation (156±30 nM vs 27±3 nM, P<0.01, Fig. 1G). These data indicate a difference between acute activation of receptor and relatively long-term action of FFAs metabolism once they have come into β-cells. The mean [Ca^2+^]i changes during above mentioned two time periods were calculated as the average Ca^2+^ levels subtracting the basal levels for the first phase increase in [Ca^2+^]i (first 3.5 min) and the second phase increase in [Ca^2+^]i (next 10 min). These parameters were used in the following results and discussion sections, when the increase in [Ca^2+^]i was analyzed in details.

### 2. Membrane Receptor-signaling Pathway and Intracellular-metabolite Signaling Pathway

**Figure 2:**
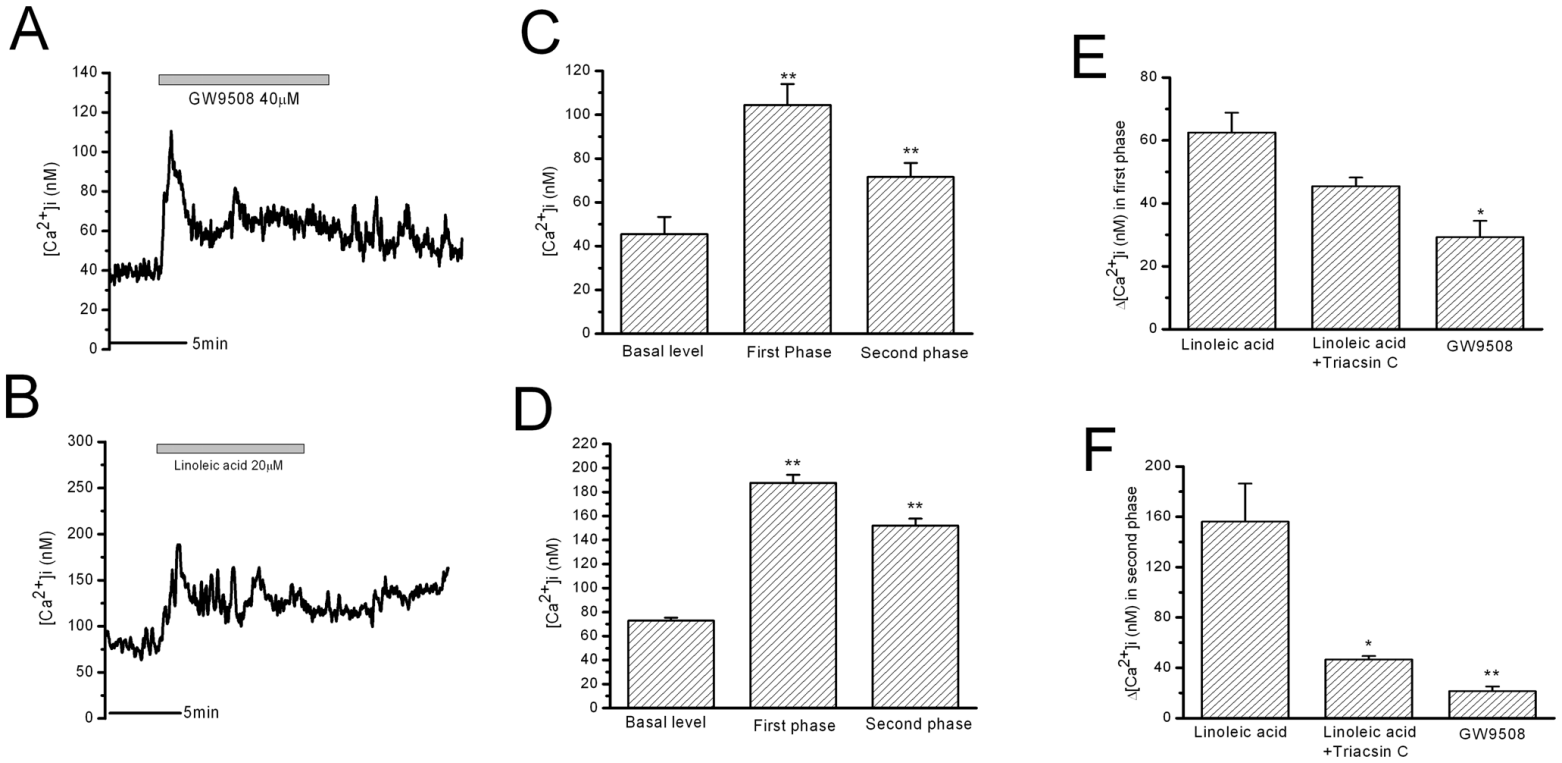
The increase in [Ca^2+^]i in rat β-cells stimulated by GW9508 and stimulated by LA after Triacsin C treatment. A: GW9508-induced increase in [Ca^2+^]i in 10 minute stimulation. B: 10 min LA-stimulated increase in [Ca^2+^]i after Triacsin C treatment. C and D: the statistical results of the maximal Ca^2+^ levels in the first phase and in the second phase of A and B, respectively. ** means P<0.01 vs basal level. E: The mean Ca^2+^ changes in the first phase. * means P<0.05 vs LA. F: The mean Ca^2+^ changes in the second phase. ** means P<0.01 vs LA (n = 7 for GW9508 and Triacsin C).

In order to observe the effect of FFA receptor-only-mediated signaling, GW9508 (a non-metabolic FFA receptor agonist from GSK Inc., UK) was applied to cause activation of FFA receptors without metabolites in rat β-cells. GW9508 (40 µM, for 10 min) stimulated the transient peak increase in [Ca^2+^]i followed by a minor plateau increase in [Ca^2+^]i (Fig. 2A and C, n = 7), highly identical to that of 2 min LA stimulation (Fig. 1B). This result suggests that the FFA receptor-mediated increase in [Ca^2+^]i should be mainly composed of the transient peak increase with minor plateau increase in [Ca^2+^]i whileas the strong and long-lasting increase in [Ca^2+^]i caused by 10 min LA stimulation may not be mediated by FFA receptor but LA metabolites in β-cells. Acylation of FFAs is the first step for FFA metabolism, which is achieved by Acyl-CoA synthetase (ACS). Triacsin C, the ACS inhibitor, was used to study the effects of LA metabolites on the increase in [Ca^2+^]i in β-cells. Triacsin C (10 µM) itself did not influence [Ca^2+^]i in β-cells during 20 min incubation. After triacsin C treatment of β-cells for 5 min, 10 min LA treatment induced the transient peak increase in [Ca^2+^]i while the strong and long-lasting second phase increase in [Ca^2+^]i was significantly suppressed (Fig. 2B and D, n = 7). The mean [Ca^2+^]i change in the first phase increase was not significantly different between the control and Triacsin C-treated group (Fig. 2E). The mean [Ca^2+^]i change in strong and long-lasting second phase increase was significantly reduced in 10 min LA stimulation by triacsin C pre-treatment (Fig. 2F).

**Figure 3:**
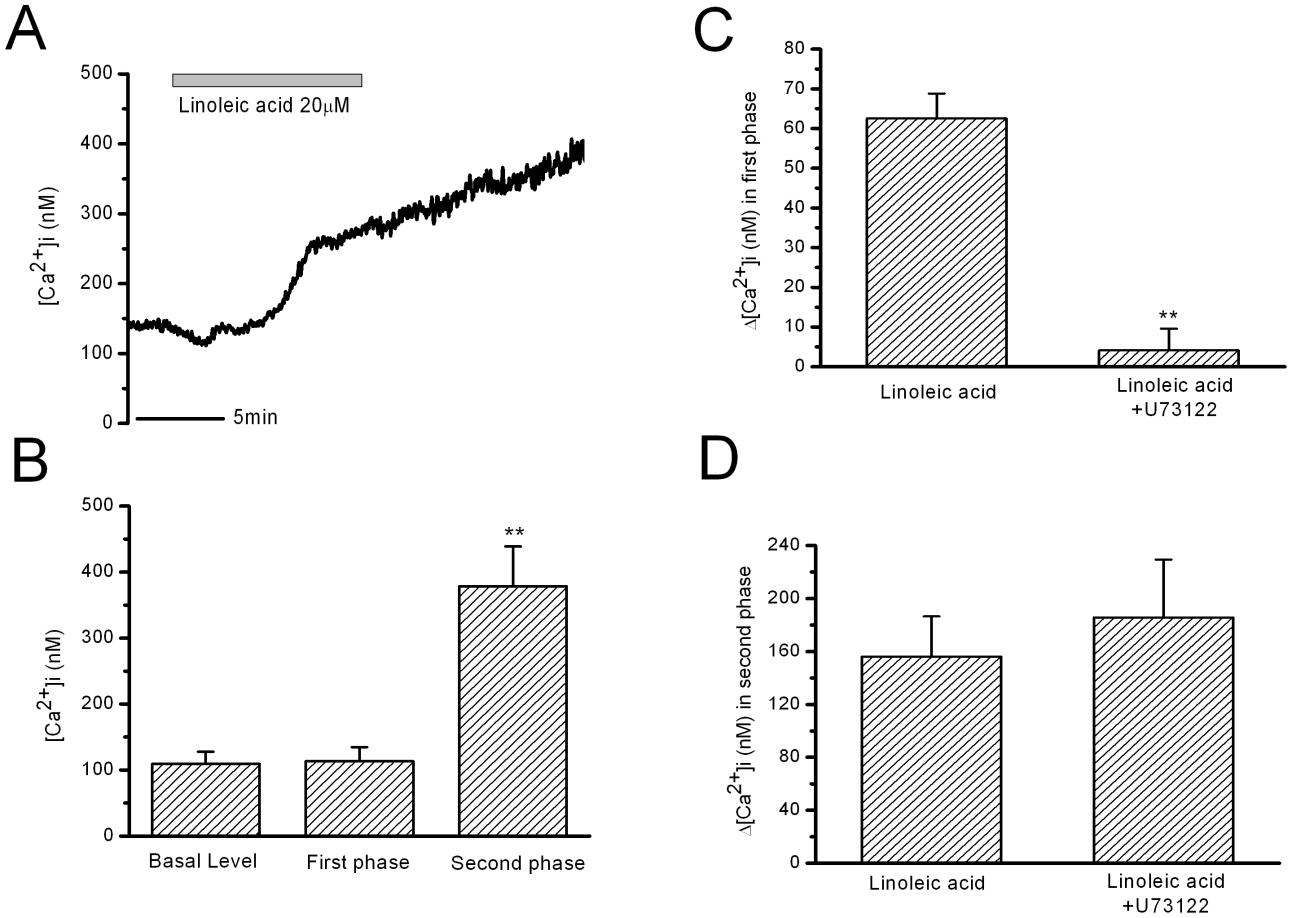
Effects of inhibiting PLC by U73122 on LA-induced increase in [Ca^2+^]i in rat β-cells. A: LA-stimulated increase in [Ca^2+^]i in U73122 treatment (10µM for 10min). B: the statistical results of the maximal Ca^2+^ levels in the first phase and in the second phase. ** means P<0.01 vs basal level and the first phase. C: The mean Ca^2+^ changes in the first phase. ** means P<0.01 vs LA. D: The mean Ca^2+^ changes in the second phase (n = 7 for U73122).

It is suggested that FFA receptor GPR40 in β-cells is coupled to phospholipase C and Inositol triphosphate (PLC/IP3) signaling pathways [16]. In the present study, we also observed that inhibition of PLC by U73122 (10 µM for 10 min) blocked the transient first phase increase in [Ca^2+^]i, but did not influence the strong and long-lasting second phase increase in [Ca^2+^]i induced by 10 min LA stimulation (Fig. 3A and B). The mean [Ca^2+^]i change in the first phase increase was almost fully eliminated by U73122 (Fig. 3C); whereas the mean [Ca^2+^]i change in the second phase was not changed by U73122 (Fig. 3D). U73122 (10 µM) itself did not significantly influence [Ca^2+^]i in β-cells.

**Figure 4:**
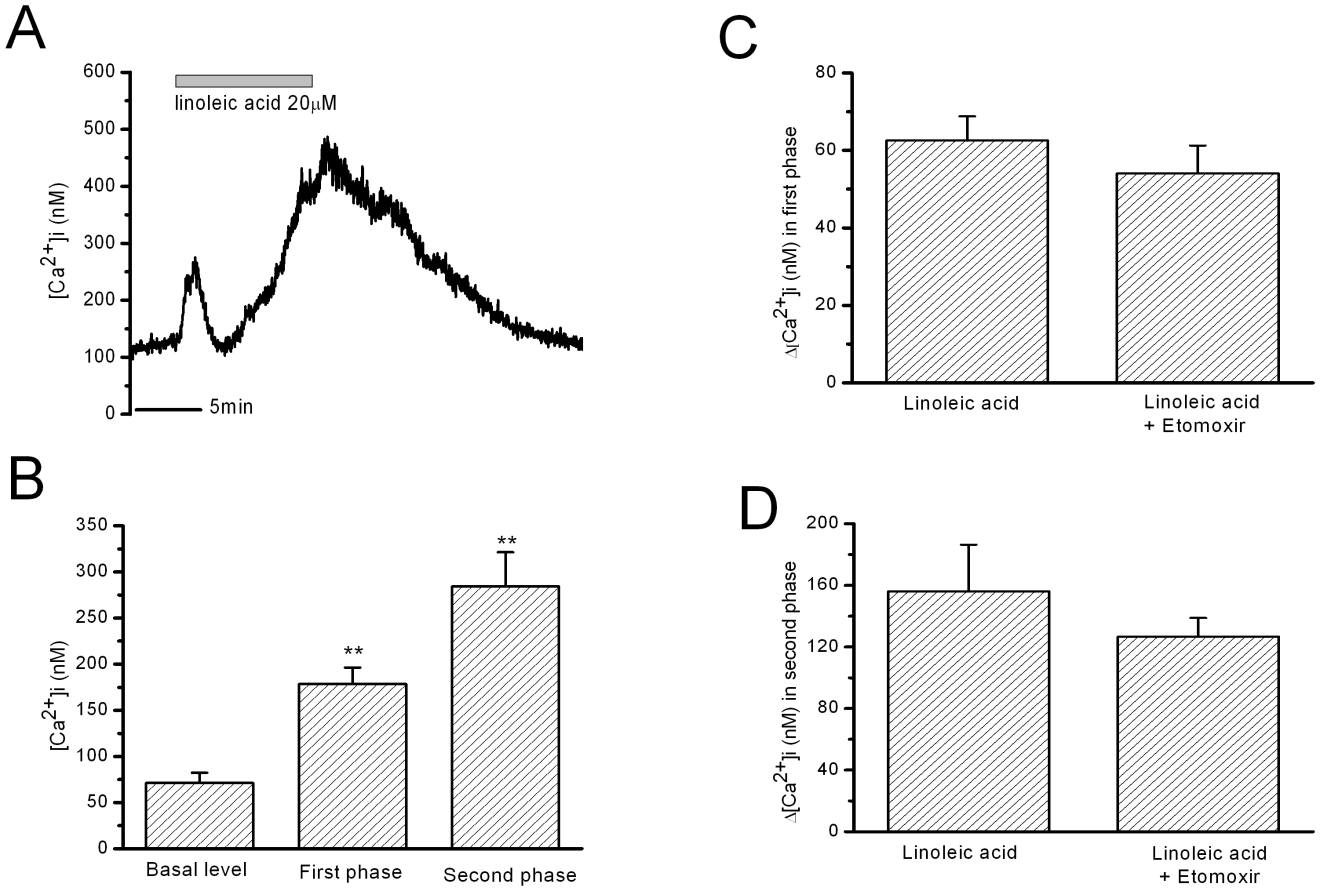
Effects of inhibiting CPT1 by etomoxir on LA-induced increase in [Ca^2+^]i in rat β-cells. A: 10 min LA-stimulated increase in [Ca^2+^]i after etomoxir treatment (0.1 mM for 10 min). B: the statistical results of the maximal Ca^2+^ levels in the first phase and in the second phase. ** means P<0.01 vs basal level. C and D: The mean Ca^2+^ changes in the first phase and in the second phase. There is no difference between LA and LA after etomoxir treatment (n = 6 for etomoxir).

Etomoxir is the carnitine palmitoyltransferase 1 (CPT1) inhibitor and inhibits the transportation of long-chain acyl-CoA into mitochondria for β-oxidation [20], [21]. However, etomoxir (0.1 mM for 10 min) did not influence the first or second phase increase in [Ca^2+^]i induced by 10 min LA stimulation (Fig. 4). Etomoxir (0.1 mM) itself did not significantly influence [Ca^2+^]i in β-cells.

### 3. The Contribution of Extracellular Calcium and Intracellular Calcium to LA-induced Increase in [Ca^2+^]i in β-cells

**Figure 5:**
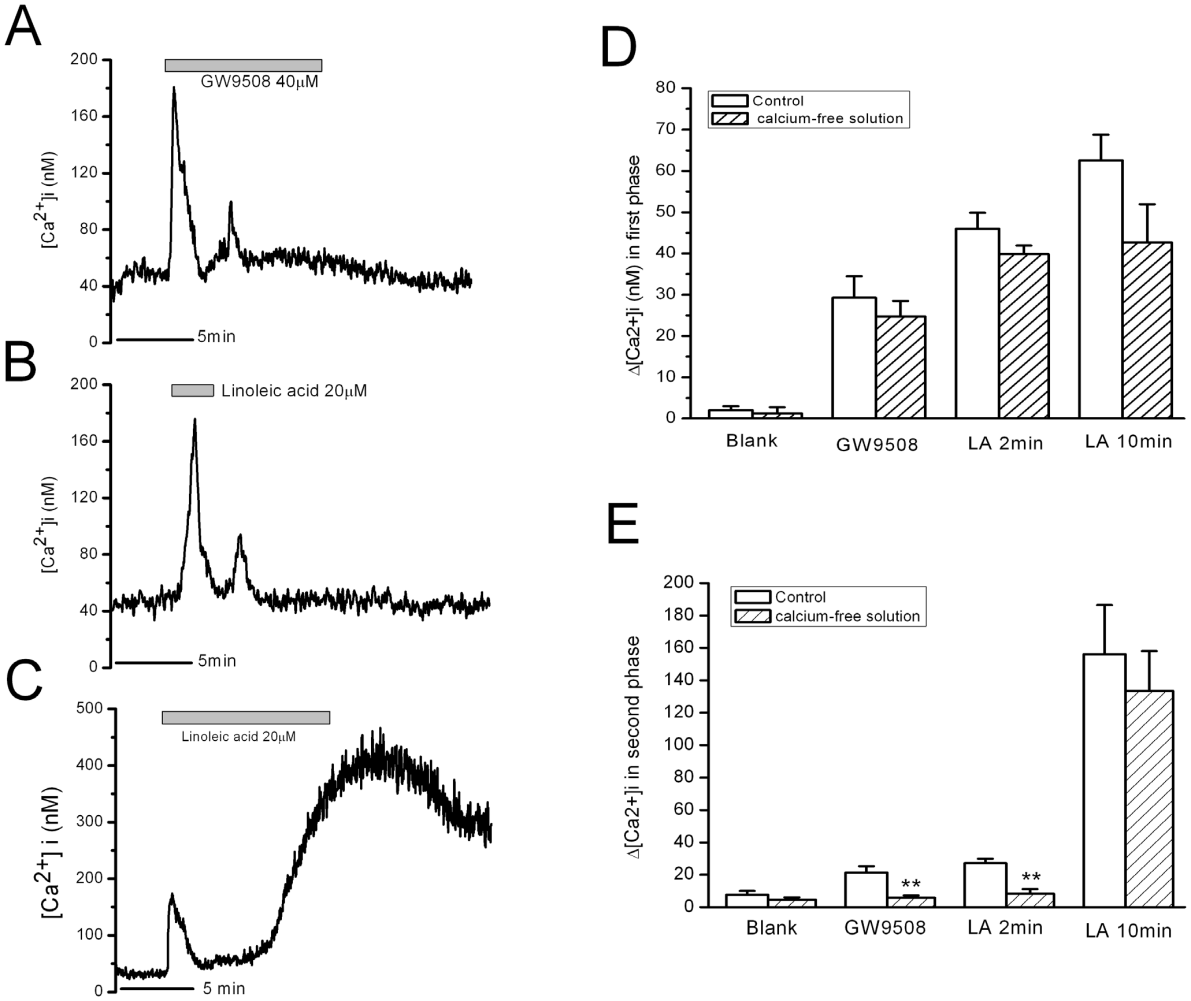
Effects of removal of extracellular Ca^2+^ on increase in [Ca^2+^]i in rat β-cells induced by GW9508, 2-min LA and 10-min LA. A–C: Increase in [Ca^2+^]i in rat β-cells induced by GW9508, 2-min LA and 10-min LA, respectively. D: The mean Ca^2+^ changes in the first phase. There is no difference between normal Ca^2+^ group and Ca^2+^-free group. E: The mean Ca^2+^ changes in the second phase. ** means P<0.01 vs normal Ca^2+^ group (n = 6 for each group without extracellular Ca^2+^).

In Ca^2+^-free bath solution, both LA and GW9508 induced the transient first phase increase in [Ca^2+^]i in β-cells. The following minor plateau increase in [Ca^2+^]i induced by 2 min LA and GW9508 was totally eliminated by removal of extracellular Ca^2+^ (Fig. 5A and B). In contrast, the strong and long-lasting increase in [Ca^2+^]i induced by 10 min LA stimulation was not affected by removal of extracellular Ca^2+^ (Fig. 5C). The mean [Ca^2+^]i changes in the first phase after LA stimulation were not affected by removal of extracellular Ca^2+^ (Fig. 5D, n = 8 for each column). The mean [Ca^2+^]i changes in the second phase were eliminated by removal of extracellular Ca^2+^ in GW9508 and 2 min LA stimulations (Fig. 5E).

**Figure 6:**
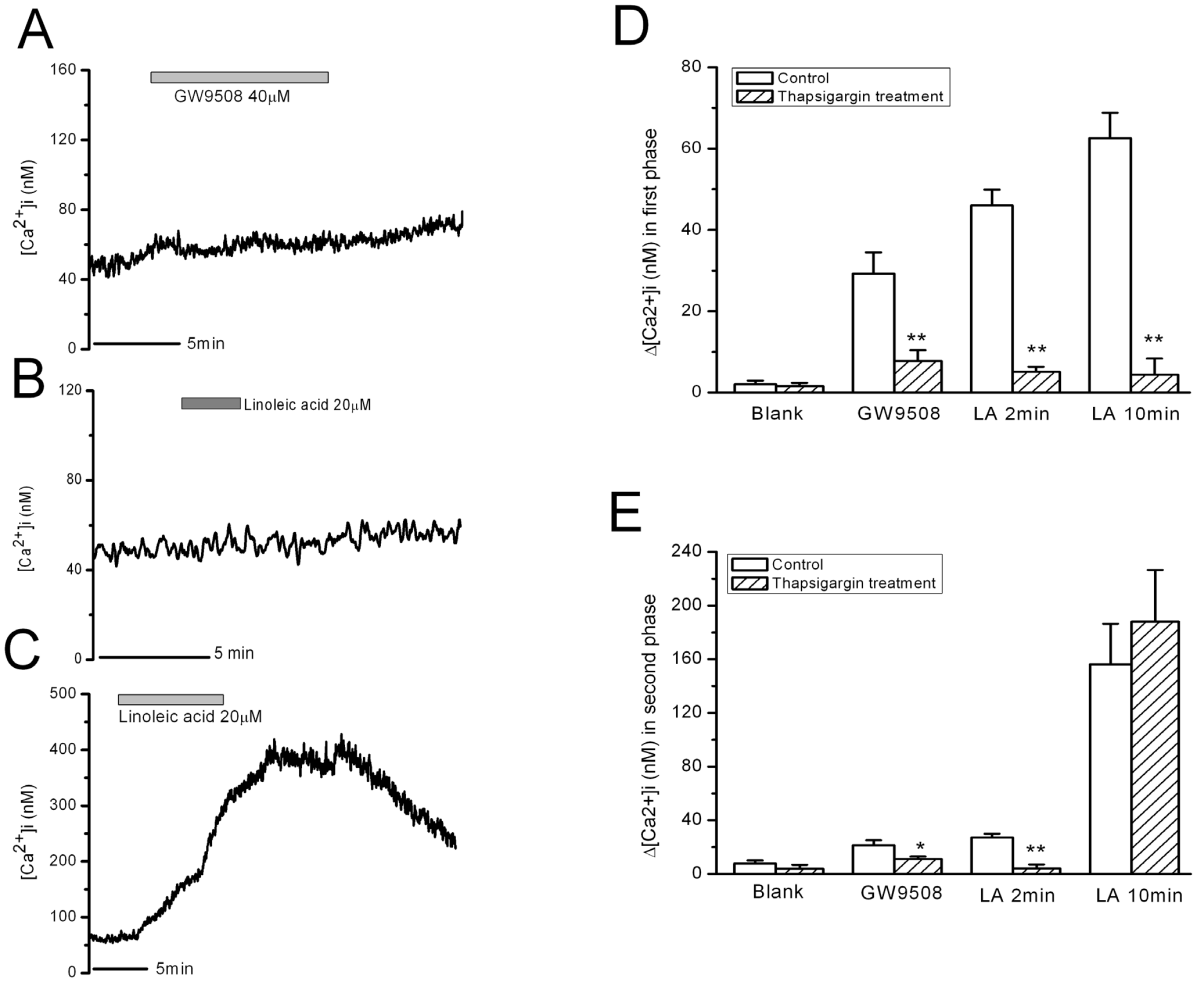
Effects of depletion of intracellular Ca^2+^ stores by thapsigargin treatment on increase in [Ca^2+^]i in rat β-cells induced by GW9508, 2-min linoleic acid (LA) and 10-min LA. A–C: Increase in [Ca^2+^]i in rat β-cells induced by GW9508, 2-min LA and 10-min LA, respectively. D: The mean Ca^2+^ changes in the first phase. ** means P<0.01 vs control group. E: The mean Ca^2+^ changes in the second phase. ** means P<0.01 vs control group (n = 8 for each group with thapsigargin treatment).

Thapsigargin is able to deplete intracellular IP3-sensitive Ca2+ pools. It induced increase in [Ca^2+^]i in β-cells, which was returned to the basal level after 20 min incubation. Depletion of intracellular IP3-sensitive Ca^2+^ pools with thapsigargin pretreatment (1 µM, 30 min) blocked the first and second phase increases in [Ca^2+^]i induced by 2 min LA and GW9508 in β-cells (Fig. 6A and B). Although the transient peak increase in [Ca^2+^]i induced by 10 min LA stimulation was blocked by thapsigargin pretreatment, the strong and long-lasting second phase increase was not significantly affected (Fig. 6C). The mean [Ca^2+^]i changes in the first and second phases are shown in Fig. 6D and E, respectively (n = 8).

**Figure 7:**
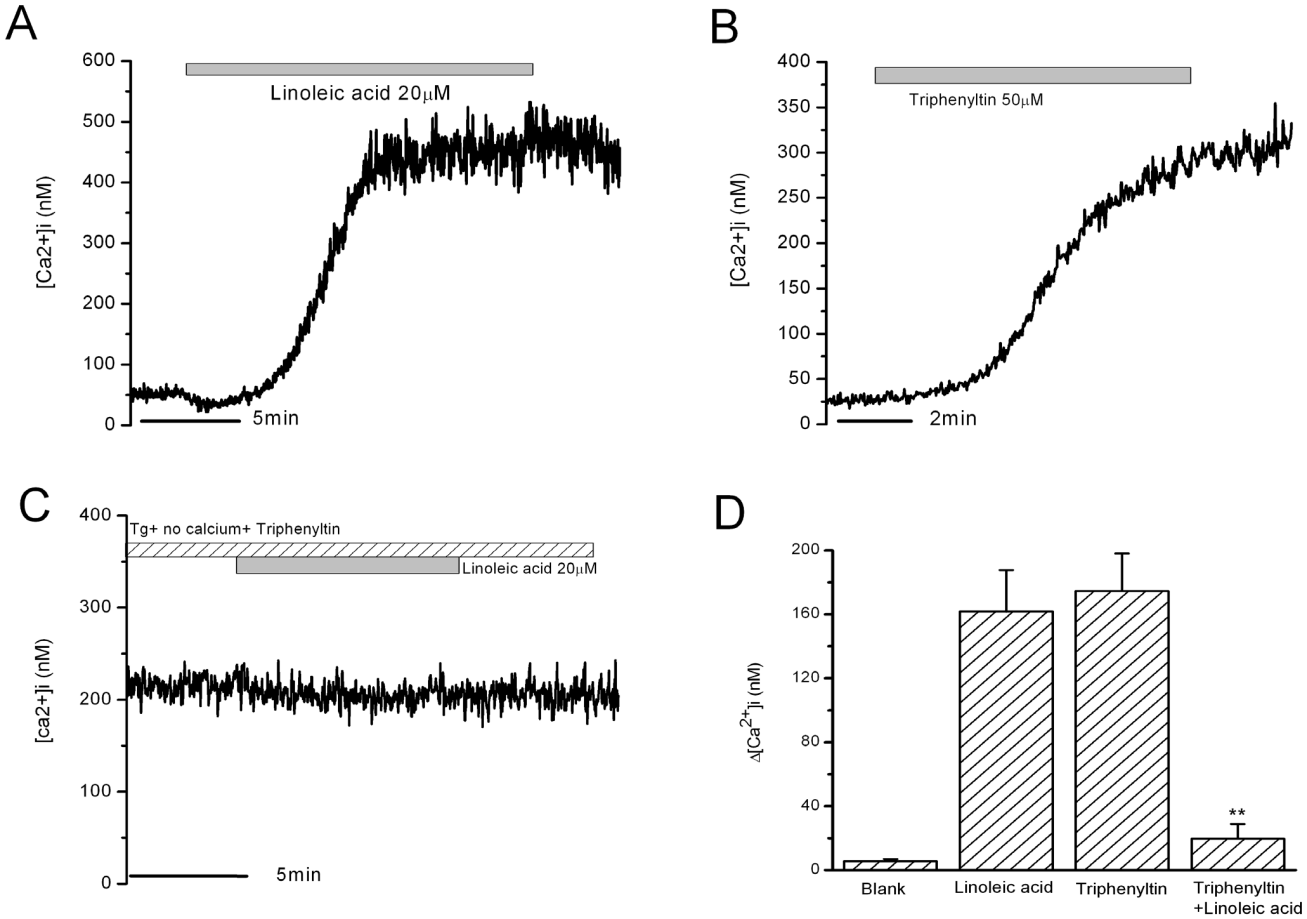
The increase in [Ca^2+^]i in rat β-cells induced by LA and triphenyltin. A: Increase in [Ca^2+^]i in rat β-cells induced by LA under the condition of thapsigargin treatment and removal of extracellular Ca^2+^. B: Increase in [Ca^2+^]i in rat β-cells induced by triphenyltin under the same condition. C: Blockade of LA-induced increase in [Ca^2+^]i after tripenyltin treatment. D: The mean Ca^2+^ change after 10 minutes stimulation. ** means P<0.0.1 vs LA and TPT (n = 9 for each group).

Neither thapsigargin pretreatment nor Ca^2+^-free bath solution totally blocked the strong and long-lasting second phase increase in [Ca^2+^]i in β-cells induced by 10 min LA stimulation. It is indicated that the thapsigargin-insensitive Ca^2+^ pool may be involved in this second phase increase in [Ca^2+^]i. This was further confirmed in Fig. 7A. LA for 10 min induced the strong and long-lasting second phase increase in [Ca^2+^]i under the condition of combination of thapsigargin pretreatment and removal of extracellular calcium. Under the same condition, triphenyltin, which was reported to induce Ca^2+^ efflux from mitochondria, induced an increase in [Ca^2+^]i similar to that induced by 10 min LA treatment (Fig. 7B). On the basis of triphenyltin pretreatment under the condition of thapsigargin pretreatment and removal of extracellular calcium, 10 min LA-induced second phase increase in [Ca^2+^]i was completely blocked (Fig. 7C). The mean [Ca^2+^]i changes after LA stimulation in different conditions were shown in Fig. 7D (n = 9).

## Discussion

In this report, we demonstrate that linoleic acid stimulated increase in [Ca^2+^]i in rat β-cells via both FFA receptor mediated pathway and intracellular metabolite mediated pathway. FFA receptor signaling pathway mediates the transient peak first phase increase and the following minor second phase plateau increase in [Ca^2+^]i in β-cells, and the intracellular metabolite signaling pathway mediates the strong and long-lasting second phase increase in [Ca^2+^]i in β-cells.

The transient peak first phase increase in [Ca^2+^]i stimulated by LA is due to FFA receptor-mediated Ca^2+^ release from endoplasmic reticulum. This is supported by the observation that the first phase increase in [Ca^2+^]i was induced by GW9508, a non-FFA agonist of FFA receptors [22]. This increase in [Ca^2+^]i was not blocked by the inhibition of LA metabolism using long-chain acyl-CoA inhibitor, Triacsin C [23]. FFA receptor GPR40 is highly expressed in pancreatic islets, and particularly in insulin-secreting β-cells [10], [19]. Activation of GPR40 leads to an increase in [Ca^2+^]i in β-cells, with a signaling pathway including Gq/11, PLC, and InsP3, triggering Ca^2+^ release from intracellular Ca^2+^ stores [16], [19]. In regard to the down-stream signaling for FFA receptor, thapsigargin was used to deplete intracellular InsP3-sensitive Ca^2+^ pools and U73122 was used to block PLC activation and down-stream InsP3 production. It was confirmed that depletion of InsP3-sensitive Ca^2+^ pool or blockade of PLC abolished the first phase increase in [Ca^2+^]i in response to LA and GW9508 in β-cells, whereas Ca^2+^-free bath solution that eliminated Ca^2+^-influx through Ca^2+^ channels did not affect the increase. These results support the view that FFA membrane receptor activation causes increase in [Ca^2+^]i in β-cells by PLC-InsP3 signaling pathways, and Ca^2+^ release from InsP3-sensitive Ca^2+^ pools is the source of the first phase increase in [Ca^2+^]i.

The following increase in [Ca^2+^]i after the transient increase in [Ca^2+^]i induced by LA is composed of two distinct components. The minor plateau increase in [Ca^2+^]i by GW9508 or short (2 min) stimulation of LA was probably mediated by calcium store-operated Ca^2+^ entry (SOCE). SOCE has been observed in many cell types, including β-cells [17], [24], [25]. It is activated by Ca^2+^ release from endoplasmic reticulum [26]. 2 min LA stimulation and GW9508 stimulated a minor sustained plateau increase in [Ca^2+^]i in β-cells, which was completely prevented by removal of extracellular calcium. It is suggested that Ca^2+^ influx is responsible for this component of [Ca^2+^]i increase. In addition, this plateau increase in [Ca^2+^]i was also blocked by elimination of Ca^2+^ release from InsP3-sensitive Ca^2+^ pools by thapsigargin treatment. This Ca^2+^ influx is therefore triggered by the calcium release from InsP3-sensitive Ca^2+^ pools and SOCE may be involved.

The major component of the second phase increase in [Ca^2+^]i induced by 10 min LA stimulation is probably mitochondrial Ca^2+^ mobilization or leakage. In Ca^2+^-free solution and depletion of intracellular Ca^2+^ pool by thapsigargin treatment, the strong and long-lasting second phase increase in [Ca^2+^]i occurred in a similar pattern to that in normal Ca^2+^ solution by 10 min LA, excluding the contribution of Ca^2+^-influx and the thapsigargin-insensitive Ca^2+^ pool. Triphenyltin induces a formation of mitochondrial permeability transition pore (PTP) and triggers Ca^2+^ release from mitochondria [27]. Triphenyltin induced an increase in [Ca^2+^]i similar to that induced by 10 min LA under the condition of combination of thapsigargin treatment and removal of extracellular calcium. In addition, LA did not induce any further increase in [Ca^2+^]i after triphenyltin treatment of β-cells. It is therefore concluded that the strong and long-lasting second phase increase in [Ca^2+^]i in rat β-cells induced by 10 min LA may be due largely to the release of mitochondria calcium store. Mitochondria contain large amounts of Ca^2+^. The free matrix calcium concentration of mitochondria is in the range 0.5–2 µM [28]. However free matrix calcium does not rise in normal circumstance because Ca^2+^ binds mostly to phosphates to form a complex [29], which provides a large reservoir of Ca^2+^ in the cell. Rapid reversibility of matrix calcium-phosphate complex can provide an increase in the free matrix Ca^2+^ concentration reaching 100-fold of the resting level [30]. The mitochondria permeability transition pore (PTP) is the major Ca^2+^ efflux pathway from mitochondria [31]. Our data suggest that LA induces mitochondrial Ca^2+^ efflux, probably through formation of PTP.

The intracellular metabolite of LA, probably Acyl-CoA, is responsible for the mitochondrial Ca^2+^ mobilization. GW9508 did not mimic the strong and long-lasting second phase increase in [Ca^2+^]i and U73122 did not block it either. Therefore, metabolites of LA, rather than FFA receptor, may trigger the signaling pathway for the mitochondrial Ca^2+^ leakage. The slow increase and recovery of this second phase increase in [Ca^2+^]i also support that the metabolism pathway may be responsible for the mitochondrial Ca^2+^ mobilization as time is required for enzyme to form and remove metabolites of LA inside the β-cells. Long-chain Acyl-CoA may be responsible or at least partially responsible for mitochondrial Ca^2+^ mobilization. FFAs inside the cells are firstly acylated by ACS and then moved to the mitochondria for β-oxidation with the help of CPT1 or processed through other pathways such as protein acylation and triglyceride formation [7]. The blockade of the strong and long-lasting second phase increase in [Ca^2+^]i by the inhibition of ACS with triacsin C, but not the inhibition of CPT1 with etomoxir, suggests that long-chain Acyl-CoA may be responsible for the metabolic effects of LA on [Ca^2+^]i changes. Long-chain acyl-CoA is an important intermediate linking fatty acid metabolism and regulation of cellular function [8]. Numerous reports have shown that long-chain acyl-CoA esters influence mitochondrial function in many cell types [32]. Long-chain acyl-CoA induces mitochondrial membrane PTP from isolated rat liver and heart mitochondria [33]–[35]. In the present study, we suggest that long-chain acyl-CoA derived from LA induces mitochondrial membrane PTP formation in rat β-cells and subsequent Ca^2+^ release. Mitochondrial PTP induces apoptosis in various types of cells [15], [36]–[38] and it may also happen in β-cells. It is known that long-term treatment with FFAs induces β-cell apoptosis [5], [6]. The present data suggest that induction of the mitochondrial PTP formation and calcium release may serve as the mechanism for FFA-induced apoptosis of β-cells. This suggestion is also supported by the finding that triacsin C deters FFA-induced β-cell apoptosis [39]. Mitochondrial dysfunction is closely associated with type 2 diabetes [40], [41]. The present study suggests that accumulation of metabolites of FFAs, probably long-chain Acyl-CoA, induce mitochondrial dysfunction and apoptosis.

In summary, both FFA membrane receptor- and intracellular metabolite-mediated signaling pathways contribute to the increase in [Ca^2+^]i in rat β-cells by LA stimulation. The patterns and Ca^2+^ sources of each pathway-linked increase in [Ca^2+^]i are different and may be associated to different β-cell functions.
